# Prevalence of Stunting and Its Associated Factors among Children of 6–59 Months in Arba Minch Health and Demographic Surveillance Site (HDSS), Southern Ethiopia: A Community-Based Cross-Sectional Study

**DOI:** 10.1155/2020/9520973

**Published:** 2020-03-21

**Authors:** Biruk Bogale, Befikadu Tariku Gutema, Yilma Chisha

**Affiliations:** ^1^Department of Public Health, Mizan Tepi University, Mizan Aman, Ethiopia; ^2^Department of Public Health, Arba Minch University, Arba Minch, Ethiopia

## Abstract

**Methods:**

The community-based cross sectional study was conducted in the Arba Minch Health and Demographic Surveillance Site, Southern Ethiopia. The simple random sampling method was used to recruit 656 mother-child pairs. Height for age *Z* score was computed using WHO Anthro version 3.2.2 software. Multivariable logistic regression model was fitted, and adjusted odds ratio (AOR) at *p* value <0.05 was used to determine statistically significant association between predictors and outcome variable.

**Result:**

The prevalence of stunting among children of 6–59 months in the study area was 47.9% (95% CI; 44.0–51.7). The likelihood of stunting was significantly higher among children who live in households with medium (AOR 2.20, 95% CI: 1.43–3.37) and poor (AOR 2.87, 95% CI: 1.72–4.81) wealth status. In addition, children who were not exclusively breast fed (AOR 1.55, 95% CI: 1.07–2.24), whose mothers had not participated in decision of major household purchases (AOR 2.27, 95% CI: 1.21–4.26), and whose mothers lacked decision on freedom of mobility (AOR 1.96, 95% CI: 1.05–3.66) were significantly stunted compared with counterparts.

**Conclusion:**

Stunting is a severe public health problem in the area. Therefore, efforts should be taken to enhance maternal empowerment, household wealth, and infant and young child feeding practice for reducing stunting among children.

## 1. Introduction

Stunting is one of the most common markers of chronic undernutrition, which is a linear growth failure or inability to attain potential height for a particular age [1, 2]. It is considered as the overall best indicator of child well-being [3]. Despite the significant achievement, the world has made towards improving nutrition and associated health burdens over recent decades, and malnutrition remains a public health threat, including stunting. Accordingly, the United Nations Nutrition report disclosed that 50.5 million children under 5 years of age are wasted and 150.8 million are stunted in 2018 [4]. Evidence shows Africa and Asia were disproportionately affected by child stunting where they accounted for more than nine out of ten of all stunted children globally [5].

In Ethiopia, stunting remains a major public health threat. The study conducted in East Belessa district of Northwest Ethiopia reported that more than half (57.7%) of under-five children were stunted [6]. Reports showed that nearly half of the children were also stunted in Northwest, Southern, and Eastern parts of the country [7–9].

Stunting has a devastating and far reaching impact on individuals and nations; it ranges from diminished cognitive, low school performance, and physical development to increased risk of degenerative diseases such as diabetes and affects the countries' economy by reducing the working capacity and productivity [1, 10, 11]. The causes of stunting are intertwined and complexes such as socioeconomic status, inadequate infant and young child feeding practices, poor sanitation and hygiene conditions, paternal education, family size, and poor maternal health and nutrition [1, 10].

There is limited evidence regarding stunting prevalence and associated factors among under-five children in the study area. Moreover, in identifying determinants of stunting, there is also paucity of evidence regarding the association of latent variables such as maternal empowerment domains with stunting. Therefore, the aim of this study was to assess the magnitude of stunting and associated factors among children of 6–59 months in Arba Minch Health and Demographic Surveillance Site (HDSS), Southern Ethiopia.

## 2. Methods and Materials

### 2.1. Study Setting and Design

The community-based cross sectional study was conducted from March to April 2019 in Arba Minch HDSS. Arba Minch HDSS located in Arba Minch Zuria District by including 9 kebeles (the smallest administration unit of Ethiopia). The Kebeles were selected from Arba Minch Zuria district by stratifying them based on the climatic zone: lowland, midland, and highland.

### 2.2. Sample Size and Sampling Technique

For estimating sample size, 45.6% prevalence of stunting was used, which was obtained from the study conducted in East Badawacho District, Southern Ethiopia [12]. In addition, 95% confidence interval, 4% margin of error, and 10% nonresponse were used. Accordingly, the calculated sample size was 656. All children aged 6 to 59 months in the site of Arba Minch HDSS were source population.

**Figure 1:**
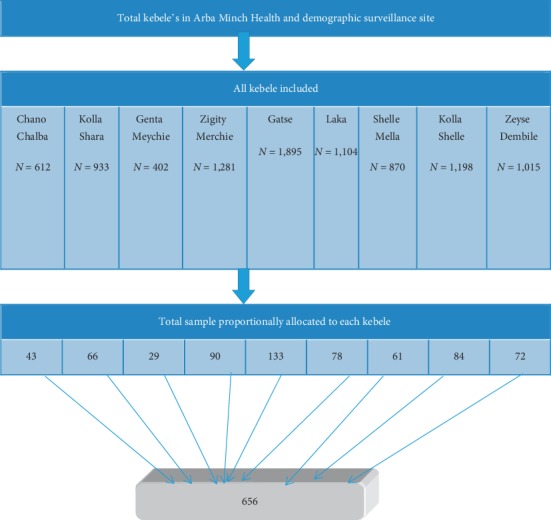
Schematic presentation of sampling technique for prevalence of stunting and its associated factors among children of 6–59 months in Arba Minch (HDSS), Southern Ethiopia.

For the selection of the study participants, the sampling frame prepared from Arba Minch HDSS dataset, which contains child date of birth (age), kebele, household identification, household head name, marital status, child name, was used. The sample was allocated to each kebele proportionally based on the number of children from 6–59 months in the kebeles. Then, study participants were randomly selected using STATA version14 from each kebele (Figure 1).

### 2.3. Data Collection Tools and Procedure

Data were collected through home to home visits using a structured and pretested interviewer-administered questionnaire. The questionnaire consisted of questions that could measure socioeconomic and demographic factors, maternal empowerment status, child and mother characteristics, and environmental health condition of the household and household food insecurity. Maternal empowerment were assessed by using survey-based Women's emPowERment index, which was validated using Demographic and Health Survey data from different African countries for the estimation of the inequalities and its effects on child health [13]. Wealth index was formed by asking assets ownership based on different variables adopted from Ethiopian Demographic and Health Survey 2016 variables [14]. Household food security status was assessed by using Household Food Insecurity Accesses Scale (HFIAS), which was developed and validated by Food and Nutrition Technical Assistance (FANTA) and again validated for Ethiopian households [15, 16]. Length was measured for children aged 6–23 months in a recumbent position, and height was measured for children aged 24 to 59 months in a standing-up position to the nearest 0.1 cm. A board with an upright wooden base and movable headpieces was used for measuring the height.

## 3. Data Quality Management

A questionnaire was translated into Amharic for common understanding. To check the consistency of the meaning, the Amharic version was translated back to English. Pretest was conducted on 5% of the households, which was not selected in actual sample in one kebele from HDSS. Supervision was made throughout data collection time by supervisors and principal investigator. Relative technical error of measurement (%TEM) was calculated during training among 10 under-five children to minimize random anthropometric measurement error. Both intra- and inter-observer variabilities were within the acceptable range.

### 3.1. Data Processing and Analysis

Data were collected using the Open Data Kit (ODK) software via a tablet (smartphone). It was transferred to Statistical Package for Social Sciences (SPSS Inc., Chicago, Illinois, USA, version 23) for analysis. Height for age *Z* score was computed using WHO Anthro version 3.2.2 software [17].

The wealth status of the households was constructed by the principal component analysis based on selected household asset and grouped in to three categorizes. The dietary diversity score were assessed using seven food groups and categorized by using the acceptable four food groups, and it is classified as adequate or not adequate [18]. The empowerment status of mothers was assessed by using six domains [13, 19], and the domains had shown significant internal consistency (with the minimum Cronbach's alpha = 0.74 for community group membership and maximum Cronbach's alpha = 0.84 for decision-making on major household purchase).

Binary logistic regression analysis was used to see the independent effect of predictors on stunting. Those variables with a *p* value of ≤0.25 during bivariable analysis were retained for multivariable analysis. The stepwise backward elimination model building procedure with likelihood ratio model comparison technique was used while building the model. Interactions and confounders were tested using beta change at cutoff point 20%. Multicollinearity was checked among predictor variables, and the variance inflation factor (VIF) was found to be less than 3. Prediction performance of predictors for stunting was checked using the ROC curve. Final model fitness status was checked by Hosmer and Lemeshow goodness of fit chi-squared test (*p* value = 0.77). Later on, adjusted odds ratio (AOR) at 95% CI was used to declare statistical significance association between dependent and independent variables.

## 4. Results

### 4.1. Demographic and Socioeconomic Characteristics

Six-hundred fifty four child mother pairs were participated in the study with the response rate of 99.7%. The mean ± SD age of child was 33.47 ± 13.74 months. The majority (65.7%) of mothers had no formal education and protestant (70.5%) in religion. From all participated household, 34.3% were food insecure and almost equal proportion of male and female children were assessed (Table 1).

### 4.2. Child Morbidity, Health Care, Feeding, and Environmental Characteristics

Ninety-four (14.4%) children had illness two weeks preceding this survey. The most common reported illness was respiratory tract infection (41.5%), followed by skin rash (27.7%) and diarrhea (26.6%). Among the total mothers participated in the study, nearly 89% had ANC visit, and more than half (56.7%) delivered their index child at home (Table 2).

### 4.3. Maternal Empowerment

Almost all mothers (98%) participated in decision-making about her and her child health care either independently or jointly with their husband. Regarding the attitude towards justification of violence, overwhelming majority of mothers were (91%) justified violence (Table 3).

### 4.4. Prevalence of Stunting

The mean (SD) height for age *Z* score of 6–59 months children was −2.01 (1.86). The prevalence of stunting among children of 6–59 months in the study area was 47.9% (95% CI; 44.0–51.7). From this, the majorities (75%) were in the age group of 24–59 months, and more than half (177) were severely stunted among those stunted children.

### 4.5. Factors Associated with Stunting among 6–59 Months Children

In bivariable logistic regression analysis, paternal education, household wealth, ANC visit, place of delivery, postnatal care, breast feeding initiation time, exclusive breast feeding, mother decision on the major household purchase, the decision on freedom of mobility, source of drinking water, and waste disposal system were significantly associated with stunting.

Based on multiple logistic regression analysis, the likelihood of stunting was more than 2 times higher among children who live in the households with medium (AOR = 2.20, 95% CI: 1.44–3.38) and poor (AOR = 2.87, 95% CI: 1.72–4.81) wealth status compared with children from rich households (Table 4). Similarly, the odds of stunting were higher among children who were not exclusively breast fed for the first 6 months (AOR = 1.55, 95% CI: 1.07–2.24) than those of exclusively breast-fed children. Those mothers who did not participate in decision of major household purchase had double risk to have stunted child (AOR = 2.27, 95% CI: 1.21–4.27), and the odds of stunting were higher among children whose mothers lack decision on freedom of mobility (AOR = 1.96, 95% CI: 1.05–3.67) (Table 4).

## 5. Discussion

This study had tried to assess the prevalence and associated factors of stunting among children of 6–59 months. Accordingly, the prevalence of stunting was 47.9% (95% CI; 44.0–51.7), which is a severe public health problem based on the WHO cutoff point [20]. The prevalence found in this study is comparable with study conducted in Libo Kemkem (49.4%), Haramaya (45.8%), and East Badawacho (45.6%), which are districts of Ethiopia [8, 12, 21]. However, the prevalence in the current study area is lower than the prevalence reported from India (51%) and Myanmar (59.4%) [22, 23]. The prevalence we found in this study is also lower than the findings reported from Merhabete (52.4%) and East Belessa (57.7%) districts in northern Ethiopia [6, 24]. This variation could be due to Agroecological and sociodemographic differences.

In comparison with the study conducted in Damot Gale (41.7%), East Gojjam (37.5%), and West Gojjam (38.3) Zones of Ethiopia, the prevalence is higher [25, 26]. It is also a much higher prevalence when compared with the findings from Yemen (42.5%), Peru (15.9%), China (17.5%), Vietnam (23.5%), and Bangladesh (41%) [27–31]. The possible explanation could be due to sociocultural difference and age category of target population. Moreover, among nine kebeles in Arba Minch HDSS, eight of them are rural kebeles and the remaining is semiurban, so that this could affect the prevalence in the area. Due to inadequate maternal and child care, stunting is more common in rural areas when compared with urban settlement [32].

In the current study, household wealth status had a significant association with stunting among 6–59 months children. It indicated that children from poorest and medium wealth status households were two times more likely to be stunted compared with children from the richest households. This is supported by the study conducted in Bangladesh, Yemen, and other studies conducted in Ethiopia [27, 28, 33]. Obviously, poverty and economic inequality hinder the financial ability for household food access, inadequate sanitation, and health care utilization, which are the major contributing factors for the development of malnutrition including stunting [34]. In contrary, household wealth had no significant association with stunting in study conducted in East Gojjam, Ethiopia [35].

Appropriate infant and young child feeding has been identified as one of key determinants of child under nutrition particularly stunting [10]. Exclusive breastfeeding has been identified as an indispensable way of providing the ideal food for the healthy growth and development of infants and children [10, 36]. As shown by this study, the odds of stunting were more common among children who were not exclusively breast-fed in the first six months. These results corroborate the findings of Fikadu et al. (2014), which found exclusive breast-feeding as a predictor of stunting in Meskan district, Southern Ethiopia [36]. This could be explained by the initiation of any type of complimentary food before six months could cause illness like diarrhea and lower respiratory tract infections, due to immature digestive and immune system of children [37].

From maternal empowerment domains, maternal decision-making on major household purchase and freedom of mobility were significantly associated with childhood stunting. Accordingly, those mothers who did not participate in financial decision of major household purchase were two times more likely to have stunted children [38]. This finding is supported by study conducted in Butajira, Ethiopia, and Benin [19, 39]. This result could suggest mothers who participate in financial decision-making could give greater proportion of resources to child-favored expenditures. It could also give mothers freedom for household expenditure towards food, which could favor dietary diversity. However, this finding is inconsistent with study conducted in Tanzania [40].

In this study, mothers who lack decision on freedom of mobility had higher odds to have stunted child. This could be best explained by mothers who lack freedom of mobility could not access health care early for sick child [41]. Moreover, they may not participate in marketing and agricultural activities, which favors food security and diversity [39]. This is in agreement with studies conducted in Butajira, Benin, and analysis of sub-Saharan demographic and health survey data [13, 19, 39]. However, freedom of mobility had no significant association with stunting in the study conducted at Jimma, Ethiopia, and Lao PDR [42, 43]. The possible reason could be due to cross cultural nature of empowerment domains from area to area [13, 44–46].

Community-based nature of the study gives representative sample among study subjects in Arba Minch HDSS, where it is useful for intervention strategies and actions. This study is not free from limitations, and recall and social desirability biases could be one of the limitations of the study. Furthermore, through intensive training, regular field supervision and measurement standardization the study were not free from the measurement error.

## 6. Conclusion

The current study showed stunting is a severe public health problem in Arba Minch HDSS, which is also based on the WHO cutoff point. Lack of maternal decision-making on major household purchase and freedom of mobility, lower household wealth status, and exclusive breast-feeding were independent predictors of stunting. Therefore, enhancing maternal decision-making and improving household economic conditions need to be considered for reducing stunting among children. It is also crucial to give due emphasis for interventions related to infant and young child feeding with special emphasis for exclusive breast feeding.

## Figures and Tables

**Table 1:** Demographics and socioeconomic characteristics of 6 to 59 months children in Arba Minch HDSS, Southern Ethiopia, 2019.

Variables	Category	Frequency	Percentage
Child sex	Male	329	50.3
	Female	325	49.7

Child age	6–24 month	193	29.5
	25–59 month	461	70.5

Mother education	No formal education	430	65.7
	Primary	154	23.5
	Secondary and above	70	10.7

Father education	No formal education	393	60.1
	Primary	172	26.3
	Secondary and above	89	13.6

Mother age	≤24 years	167	25.5
	25–34 years	409	62.5
	≥35 years	78	11.9

Family size	≤4	174	26.6
	5-6	267	40.8
	≥7	213	32.6

Child birth order	1^st^	140	21.4
	2^nd^ and 3^rd^	245	37.5
	4^th^ and above	269	41.1

Religion	Protestant	461	70.5
	Orthodox	193	29.5

Mother occupation	House wife	504	77
	Farmer	125	19.1
	Merchant	17	2.6
	Govt. or private employee	8	1.3

Household wealth	Rich	164	25.1
	Medium	326	49.8
	Poor	164	25.1

Food security	Food secured	430	65.7
	Food insecured	224	34.3

**Table 2:** Morbidity, health care, feeding, and environmental characteristics of study participant in Arba Minch HDSS, Southern Ethiopia, 2019.

Variables	Category	Frequency	Percentage
Child birth outcome	Single	642	98.2
	Multiple	12	1.8

Child illness 2 weeks preceding survey	No	560	85.6
	Yes	94	14.4

Number of ANC follow up	No follow up	73	11.2
	Once	10	1.5
	Twice	72	11
	Three times	228	34.9
	Four times	271	41.4

Place of delivery	Home	371	56.7
	Health facility	283	43.3

Postnatal care	No	203	31
	Yes	451	69

Breast feeding	Yes	625	95.6
	No	29	4.4

Bottle feeding	Yes	393	60.1
	No	261	39.9

Complimentary feeding practice	Early	254	38.8
	Timely	101	15.4
	Late	299	45.7

Dietary diversity	≥4 food groups	324	49.5
	<4 food group	330	50.5

Source of drinking water	Improved	539	82.4
	Unimproved	115	17.6

Latrine availability	Yes	622	95.1
	No	32	4.9

Waste disposal	Burning	243	37.2
	Open field	175	26.8
	Pit	236	36.1

**Table 3:** Maternal decision-making based on domains of women empowerment among those who have children from 6–59 month in Arba Minch HDSS, Southern Ethiopia, 2019.

Domains of women empowerment	Frequency	Percentage
By her/jointly with husband for her healthcare	644	98.5
By her/jointly with husband on child health care	644	98.5
By her/jointly with husband on major household purchase	591	90.4
By her/jointly with husband on her mobility.	594	90.8
Justify attitude toward violence	594	90.8
Participate in community group membership	491	75.1

**Table 4:** Bivariable and multivariable logistic regression analysis result for factors associated with stunting among 6–59 month children in Arba Minch HDSS, southern Ethiopia, 2019.

Characteristics	Category	Stunting		COR	AOR (95% CI)
		Yes (%)	No (%)		
Mother education	Secondary and above	26 (37.0)	44 (63.0)	Ref.	
	Primary	48 (31.0)	106 (69.0)	0.77	0.73 (0.40–1.36)
	No formal education	239 (55.6)	191 (44.4)	2.12	1.13 (0.60–2.10)

Father educational status	Secondary and above	30 (33.7)	59 (66.3)	Ref.	
	Primary	68 (39.5)	104 (60.5)	1.28	0.90 (0.44–1.85)
	No formal education	215 (54.7)	178 (45.3)	2.37	1.07 (0.59–1.99)

Household wealth index	Rich	47 (28.6)	117 (71.4)	Ref.	
	Medium	165 (50.6)	161 (49.4)	2.55	**2.20 (1.44–3.38)** ^*∗∗*^
	Poor	101 (61.5)	63 (38.5)	3.99	**2.87 (1.72–4.81)** ^*∗∗*^

ANC visit	Yes	267 (45.9)	314 (54.1)	Ref.	
	No	46 (63.0)	27 (37.0)	2.00	1.15 (0.63–2.08)

Place of delivery	Health facility	112 (39.6)	171 (60.4)	Ref.	
	Home	201 (54.1)	170 (45.9)	1.80	0.92 (0.60–1.41)

Postnatal care	Yes	190 (42.1)	261 (57.9)	Ref.	
	No	123 (60.6)	80 (39.4)	2.11	0.67 (0.40–1.14)

Breast feeding initiation time	Within 1 hour	127 (40.9)	183 (59.1)	Ref.	
	After 1 hour	186 (54.0)	158 (46.0)	1.70	1.24 (0.85–1.81)

Exclusive breast feeding	Yes	152 (38.7)	241 (61.3)	Ref.	
	No	161 (61.7)	100 (38.3)	2.55	**1.55 (1.07–2.24)** ^*∗*^

Dietary diversity	≥ 4 food groups	146 (45.0)	178 (55.0)	Ref.	
	<4 food groups	167 (50.6)	163 (49.4)	1.25	0.84 (0.54–1.29)

Decision on major household purchase	Her/jointly with husband	266 (45.0)	325 (55.0)	Ref.	
	Husband only	47 (74.6)	16 (25.4)	3.59	**2.27 (1.21–4.27)** ^*∗*^

Decision on freedom of mobility	Her/jointly with husband	271 (45.6)	323 (54.4)	Ref.	
	Husband only	42 (70.0)	18 (30.0)	2.78	**1.96 (1.05–3.67)** ^*∗*^

Household food security	Secure	196 (45.6)	234 (54.4)	Ref.	
	Insecure	117 (52.2)	107 (47.8)	1.30	1.24 (0.83–1.86)

Source of drinking water	Improved	242 (44.9)	297 (55.1)	Ref.	
	Unimproved	71 (61.7)	44 (38.3)	1.98	1.12 (0.70–1.81)

Waste disposal	Burning	105 (43.2)	138 (56.8)	Ref.	
	Open field	102 (58.3)	73 (41.7)	1.83	1.16 (0.73–1.83)
	Pit	106 (44.9)	130 (55.1)	1.07	0.80 (0.54–1.19)

p value of ^*∗*^<0.05 and ^*∗∗*^<0.01, COR crude odds ratio, AOR adjusted odds ratio.

## Data Availability

Data will be available upon reasonable request from the corresponding author.

## Abbreviations

ANC: Antenatal care
AOR: Adjusted odds ratio
COR: Crude odds ratio
HDSS: Health and demographic surveillance site
WHO: World Health Organization.

## References

[1] Dewey K. G., Begum K. (2011). Long-term consequences of stunting in early life. *Maternal & Child Nutrition*.

[2] Reinhardt K., Fanzo J. (2014). Addressing chronic malnutrition through multi-sectoral, sustainable approaches: a Review of the causes and consequences. *Frontiers in Nutrition*.

[3] de Onis M., Branca F. (2016). Childhood stunting: a global perspective. *Maternal & Child Nutrition*.

[4] Development Initiatives (2018). *2018 Global Nutrition Report: Shining a Light to Spur Action on Nutrition*.

[5] FAO (2018). *The State of Food Security and Nutrition in the World 2018. Building Climate Resilience for Food Security and Nutrition*.

[6] Fentahun W., Wubshet M., Tariku A. (2016). Undernutrition and associated factors among children aged 6–59 months in East Belesa district, Northwest Ethiopia: a community based cross-sectional study. *BMC Public Health*.

[7] Tariku A., Woldie H., Fekadu A., Adane A. A., Ferede A. T., Yitayew S. (2016). Nearly half of preschool children are stunted in Dembia district, Northwest Ethiopia : a community based cross- sectional study. *Archives of Public Health*.

[8] Yisak H., Gobena T., Mesfin F. (2015). Prevalence and risk factors for under nutrition among children under five at Haramaya district, Eastern Ethiopia. *BMC Pediatrics*.

[9] Birhanu Y. W., Endris A. Y. (2015). Predictors of poor anthropometric status among children under two years of age in Gamo Gofa zone, Southern Ethiopia, 2015; cross-sectional study. *Epidemiology (Sunnyvale)*.

[10] Stewart C. P., Iannotti L., Dewey K. G., Michaelsen K. F., Onyango A. W. (2013). Contextualising complementary feeding in a broader framework for stunting prevention. *Maternal & Child Nutrition*.

[11] Martorell R., Horta B. L., Adair L. S. (2010). Weight gain in the first two years of life is an important predictor of schooling outcomes in pooled analyses from five birth cohorts from low- and middle-income countries. *The Journal of Nutrition*.

[12] Betebo B., Ejajo T., Alemseged F., Massa D. (2017). Household food insecurity and its association with nutritional status of children 6–59 Months of age in East Badawacho district , south Ethiopia. *Journal of Environmental and Public Health*.

[13] Ewerling F., Lynch J. W., Victora C. G. (2017). The SWPER index for women’s empowerment in Africa: development and validation of an index based on survey data. *The Lancet Global Health*.

[14] Central Statistical Agency (CSA) (2016). *Ethiopia Demographic and Health Survey 2016, Addis Ababa, Ethiopia, and Rockville*.

[15] Coates J., Bilinsky P., Coates J. (2007). *Household Food Insecurity Access Scale (HFIAS) for Measurement of Food Access : Indicator Guide Version 3*.

[16] Gebreyesus S. H., Lunde T., Mariam D. H., Woldehanna T., Lindtjørn B. (2015). Is the adapted Household Food Insecurity Access Scale (HFIAS) developed internationally to measure food insecurity valid in urban and rural households of Ethiopia?. *BMC Nutrition*.

[17] Group WHOMGRS, Onis M. (2006). WHO Child Growth Standards based on length/height, weight and age. *Acta Paediatrica*.

[18] World Health Organization Indicators for assessing infant and young child feeding practices: part 1: definitions.

[19] Egata A. D., Deressa W. (2018). Maternal disempowerment and sever food insecurity as determinants of undernutrition among 6–36 month old children in Gurage zone. *Southern Ethiopia: Case-Control Study*.

[20] Cashin K., Oot L. (2018). Guide to anthropometry: a practical tool for program planners, managers, and implementers. *Food and Nutrition Technical Assistance III Project (FANTA)/FHI 360*.

[21] Geberselassie S. B., Abebe S. M., Melsew Y. A., Mutuku S. M., Wassie M. M. (2018). Prevalence of stunting and its associated factors among children 6–59 months of age in Libo-Kemekem district, Northwest Ethiopia; a community based cross sectional study. *PLoS One*.

[22] Sarkar S. (2016). Cross-sectional study of child malnutrition and associated risk factors among children aged under five in West Bengal, India. *International Journal of Population Studies*.

[23] Hein A. K., Hong S. A., Puckpinyo A., Tejativaddhana P. (2019). Dietary diversity, social support and stunting among children aged 6–59 months in an internally displaced persons camp in Kayin state, Myanmar. *Clinical Nutrition Research*.

[24] Abeway S., Gebremichael B., Murugan R., Assefa M., Adinew Y. M. (2018). Stunting and its determinants among children aged 6–59 months in Northern Ethiopia: a cross-sectional study. *Journal of Nutrition and Metabolism*.

[25] Abera L., Dejene T., Laelago T. (2018). Magnitude of stunting and its determinants in children aged 6–59 months among rural residents of Damot Gale district; Southern Ethiopia. *BMC Research Notes*.

[26] Motbainor A., Worku A., Kumie A. (2015). Stunting is associated with food diversity while wasting with food insecurity among underfive children in East and West Gojjam zones of Amhara region, Ethiopia. *PLoS One*.

[27] De Souza L. R. (2017). Correlates of child undernutrition in Yemen. *Bandung: Journal of the Global South*.

[28] Sarma H., Khan J. R., Asaduzzaman M. (2017). Factors influencing the prevalence of stunting among children aged below five years in Bangladesh. *Food and Nutrition Bulletin*.

[29] Wang J., Wang H., Chang S. (2015). The influence of malnutrition and micronutrient status on anemic risk in children under 3 years old in poor areas in China. *PLoS One*.

[30] Chávez-Zárate A., Maguiña J. L., Quichiz-Lara A. D., Zapata-Fajardo P. E., Mayta-Tristán P. (2019). Relationship between stunting in children 6 to 36 months of age and maternal employment status in Peru: a sub-analysis of the Peruvian Demographic and Health Survey. *PLoS One*.

[31] Chuc D. V., Hung N. X., Trang V. T., Linh D. V., Khue P. M. (2019). Nutritional status of children aged 12 to 36 months in a rural district of Hungyen Province, Vietnam. *BioMed Research International*.

[32] Ahmed A., Abdulahi A., Shab-bidar S., Rezaei S. (2014). Nutritional status of under five children in Ethiopia : a systematic review and meta-analysis. *Ethiopian Journal of Health Sciences*.

[33] Mohammed S. H., Habtewold T. D., Tegegne B. S. (2019). Dietary and non-dietary determinants of linear growth status of infants and young children in Ethiopia: hierarchical regression analysis. *PLoS One*.

[34] United Nations Children’s Fund (1990). Strategy for improved nutrition of children and women in developing countries. *A UNICEF Policy Review [Microform]*.

[35] Alemu Z. A., Ahmed A. A., Yalew A. W., Birhanu B. S., Zaitchik B. F. (2017). Individual and community level factors with a significant role in determining child height-for-age *Z* score in East Gojjam Zone, Amhara Regional State, Ethiopia: a multilevel analysis. *Archives of Public Health*.

[36] Fikadu T., Assegid S., Dube L. (2014). Factors associated with stunting among children of age 24 to 59 months in Meskan district, Gurage Zone, South Ethiopia: a case-control study. *BMC Public Health*.

[37] Kramer M. S., Kakuma R. (2012). Optimal duration of exclusive breastfeeding. *Cochrane Database of Systematic Reviews*.

[38] Cunningham K., Ploubidis G. B., Menon P. (2015). Women’s empowerment in agriculture and child nutritional status in rural Nepal. *Public Health Nutrition*.

[39] Alaofè H., Zhu M., Burney J., Naylor R., Douglas T. (2017). Association between women’s empowerment and maternal and child nutrition in Kalalé district of Northern Benin. *Food and Nutrition Bulletin*.

[40] Ross-Suits H. M. (2010). Maternal autonomy as a protective factor in child nutritional outcome in Tanzania.

[41] Bhagowalia P., Quisumbing A. R. (2012). *What Dimensions of Women’s Empowerment Matter Most for Child Nutrition ? Evidence Using Nationally Representative Data from Bangladesh*.

[42] Abate K. H., Belachew T. (2017). Women’s autonomy and men’s involvement in child care and feeding as predictors of infant and young child anthropometric indices in coffee farming households of Jimma Zone, South West of Ethiopia. *PLoS One*.

[43] Kamiya Y., Nomura M., Ogino H., Yoshikawa K., Siengsounthone L., Xangsayarath P. (2018). Mothers’ autonomy and childhood stunting: evidence from semi-urban communities in Lao PDR. *BMC Womens Health*.

[44] Asaolu I. O., Halimatou A., Gunn J. K. L. (2018). Measuring women’s empowerment in Sub-Saharan Africa: exploratory and confirmatory factor analyses of the demographic and health surveys. *Frontiers in Psychology*.

[45] Richardson R. A. (2018). Measuring women’s empowerment: a need for context and caution. *The Lancet Global Health*.

[46] Miedema S. S., Haardörfer R., Girard A. W., Yount K. M. (2018). Women’s empowerment in East Africa: development of a cross-country comparable measure. *World Development*.

